# Adding dynamic consent to a longitudinal cohort study: A qualitative study of EXCEED participant perspectives

**DOI:** 10.1186/s12910-021-00583-w

**Published:** 2021-02-09

**Authors:** Susan E. Wallace, José Miola

**Affiliations:** 1 Department of Health Sciences, University of Leicester, Leicester, UK; 2 School of Law, University of Leeds, Leeds, UK

**Keywords:** Dynamic consent, Data protection, Biomedical research, Longitudinal cohort studies, Biobank

## Abstract

**Background:**

Dynamic consent has been proposed as a process through which participants and patients can gain more control over how their data and samples, donated for biomedical research, are used, resulting in greater trust in researchers. It is also a way to respond to evolving data protection frameworks and new legislation. Others argue that the broad consent currently used in biobank research is ethically robust. Little empirical research with cohort study participants has been published. This research investigated the participants’ opinions of adding a dynamic consent interface to their existing study.

**Methods:**

Adult participants in the Extended Cohort for E-health, Environment and DNA (EXCEED) longitudinal cohort study who are members of the EXCEED Public and Participant Engagement Group were recruited. Four focus groups were conducted and analysed for thematic content. Discussion topics were derived from a review of the current literature on dynamic consent.

**Results:**

Participants were in favour of many aspects of a dynamic consent interface, such as being able to update their information, add additional data to their records and choose withdrawal options. They were supportive provided it was simple to use and not intrusive. Participants expressed a markedly high level of trust in the study and its investigators and were unanimously happy with their current participation. No strong support was found for adding a dynamic consent interface to EXCEED.

**Conclusions:**

Trust in the study researchers was the strongest theme found. Openness and good data security were needed to retain their trust. While happy to discuss dynamic consent, participants were satisfied with the current study arrangements. There were indications that changing the study might unnecessarily disturb their trust. This raised the question of whether there are contexts where dynamic consent is more appropriate than others. This study was limited by the small number of participants who were committed to the study and biased towards it. More research is needed to fully understand the potential impact of adding a dynamic consent interface to an existing cohort study.

## Background

The Extended Cohort for E-health, Environment and DNA (EXCEED) is led by the University of Leicester, in partnership with University Hospitals of Leicester NHS Trust and in collaboration with Leicestershire Partnership NHS Trust, local general practices and smoking cessation services[1]. Its aim is to develop a greater understanding of the genetic, environmental, and lifestyle-related causes of health and disease. Participants are aged between 30 and 69 years and were recruited through general practices in Leicester City, Leicestershire and Rutland, UK. There are over 10,000 participants. Like many cohorts, they are mostly older and female. A small proportion of participants (5%) report Asian and Asian British ethnicity [1], and future recruitment will focus on increasing this percentage.

Like many longitudinal cohort studies, EXCEED is looking for ways to make best scientific use of this precious resource, while adhering to its stated mission and the consents given by its participants. With changes to the legal frameworks and legislation surrounding data protection (i.e. the European General Data Protection Regulation (GDPR)[2] and the UK Data Protection Act 2018[3]), it is necessary to ensure that EXCEED continues to fall within agreed legal structures. Equally, as a consequence of the landmark judgement in *Montgomery v Lanarkshire Health Board*, in 2015[4], discussions around consent are now changing, “…redefin[ing] the entire basis of the doctor-patient relationship in the eyes of the law…”[5]. It is no longer sufficient to provide information based simply on what the medical professional believes they should provide. Disclosure must focus on what information the *patient or participant* wishes to receive to make an informed decision. This shift in thinking has also penetrated the research sphere. It has given EXCEED the opportunity to explore new approaches for involving participants in discussions regarding how they wish to interact with the study. One approach suggested was to design a dynamic consent interface.

## Background

Dynamic Consent has two accepted meanings in the literature. First, it is, “…a new approach for engaging individuals about the use of their personal information”[6]. This reflects the changing attitudes towards consent, away from the traditional paternalistic model and towards a more patient/participant-centred model. It also reflects the continuing concern about ‘broad’ consent in biobanking[7, 8]. Under broad consent individuals can agree to the use of their samples and associated data for future unknown research projects, reassured that appropriate governance mechanisms, including ethics review, are in place. This has been used successfully by studies such as UK Biobank[9]. However, many have questioned whether a broad consent continues to be a truly informed consent[6, 10]. A recent study, “…suggest[s] that preferences collected during the initial consent process do not dependably predict long term opinions of biobank participants”[11]. Researchers are now investigating technological approaches or ‘participant-centric initiatives’ [12] in biobanking to overcome perceived shortcomings in broad consent.

This leads to dynamic consent’s second meaning, “… a personalised communication interface to enable greater participant engagement in clinical and research activities” tailored to fit the research study[6]. Through an interface, researchers can provide information about current and proposed research activities to consented participants. Participants could see their consent preferences in one place and modify them if they wish or withdraw from the study, at their own convenience. An interface could enable participants to state their preferences to be re-contacted to complete a questionnaire for an existing study, or to join new studies. There are several perceived advantages. A dynamic consent interface could allow better communication between researchers and participants[6]. Public and participant trust could be improved through greater transparency and accountability[6, 13]. Using an interface could improve scientific literacy[6]. Interfaces can be changed to reflect changing legal requirements [13]. Because it is an online tool, it could be a way to improve inclusivity and remove barriers [14]. It has been suggested that dynamic consent can improve trust and encourage engagement in less well represented communities, “…by promoting improved understanding of the biobank and its research outputs, and a stronger sense of collaboration between donors and researchers”[15].

Those more sceptical of patient-centric interfaces question the rush to discard broad consent[16]. Broad consent, in their view, also respects the autonomy of participants[16]. Participants, by accepting broad consent, have shown that they are comfortable with others making decisions on their behalf regarding future use of data and samples. Being asked for their opinion may make some question their own competence to participate[16]. Asking for consent for every new study could make those new options appear trivial[16]. Patient-centric interfaces must be kept secure and up to date for an unknown length of time, which could have cost and staffing implications. Some groups, such as the elderly or disadvantaged, may not be willing or able to engage in a technology-based consent process. This could be due to a lack of desire or understanding, or access to infrastructure[17, 18].

The legal framework, which provides that researchers must obtain the informed consent of participants before they may be included in a study, does not preclude the giving of broad consent, and nor is it likely to do so at any time soon. This is true both of the GDPR (and it should be noted that at the time of writing that given Brexit there is no certainty about the future application in the UK of the GDPR), and indeed of the common law. That said, it is the latter that provides an interesting perspective. The landmark case of *Montgomery* v *Lanarkshire Health Board* mentioned above directly connects the concept of informed consent with autonomy, and indeed the courts have been moving towards such an explicit linkage for some time[19]. In this regard, the proponents of dynamic consent may well opine with some justification that dynamic consent interfaces are very much consistent with the view of the doctor-patient relationship imagined by the Supreme Court. Thus, “patients are now widely regarded as persons holding rights, rather than as the passive recipients of the care of the medical profession. They are also widely treated as consumers exercising choices”[4]. In this atmosphere, then, there will inevitably be pressure to move away from broad consent, and towards a dynamic consent model. *Montgomery*, despite being a case about the doctor-patient relationship, is still highly relevant to the research context. This is because the Supreme Court’s judgment did not limit itself to the facts of the case. Rather, the judgment is deliberately wide-ranging and concerns itself with medical law as a whole, cementing autonomy as central to any and all cases [5]. Moreover, it concentrated on the relationship and balance of power between medical professionals and patients in a manner which, if anything, will be even more applicable in a researcher-participant interaction than it is in the doctor-patient relationship given the importance it placed on bodily integrity and the ability of patients/participants to exercise choice based on the information that *they are entitled to*. This is a view shared by McHale, who argues that the new standard of disclosure imposed by *Montgomery* will be “impossible to ignore” in the research context, particularly given the emphasis on autonomy and the fact that a research participant is “an individual who is a participant in clinical research can be seen as acting fundamentally in the public interest and for the public benefit”[20]. It should also be noted that not only was the decision more of an evolution than revolution, in that the courts had been moving in its direction for a while, but it was also consistent with the critiques of medical law that existed in the academic literature[5, 19].

Researchers have been exploring dynamic consent with different communities. Examples include rare disease communities[21, 22], in studies using electronic medical records[17, 23], and, like EXCEED, ‘healthy volunteer’ studies[24–27]. Details are limited on how healthy volunteer studies have implemented a dynamic consent approach. A recent survey reported that five large-scale research cohorts, at the time of writing, had set up interfaces to implement a dynamic consent approach. The authors see “dynamic-informed consent” as comprising dynamic permissions, dynamic education, and dynamic preferences[28]. Within each of these, there are specific actions such as the ability to select whether to receive individual research results as a dynamic preference[28]. Each of the five studies has implemented some of these activities. For example, the RUDY (Rare UK Diseases of Bone, Joints and Blood Vessels) study, has an on-line interface where participants self-report data about themselves and their condition, and actively choose the research for which their data and samples can be used[21, 22]. The Cooperative Health Research in South Tyrol (CHRIS) study is a population-based longitudinal study “… [investigating] the genetic basis of common chronic conditions associated with human ageing, and their interaction with life-style and environmental factors in the general population of South Tyrol”[25]. Like EXCEED, the CHRIS study allows for the return of individual genetic findings. Its participants can choose, through their interface, whether to receive results. None of the five studies had implemented all of the elements for dynamic-informed consent. The only element all had included was the ability to withdraw[28]. This suggests that achieving full dynamic-informed consent is complex and to be successful, a step-by-step approach was needed.

After discussions with the EXCEED Scientific Committee, the hypothesis was formed that participants would be enthusiastic about EXCEED adding a dynamic consent interface. However, any system would need be accepted by the EXCEED participants and engagement was needed to identify what components they would want included. It was also clear that there were enough questions around dynamic consent that our hypothesis would need to be confirmed through a research study as opposed to simply implementing a new system. Focus groups were chosen as they allowed us to explore the shared ideas and opinions of the participants who would be using an interface. The following research questions were identified: a) what do our participants think about the concept of dynamic consent and b) what characteristics of a dynamic consent interface did EXCEED participants believe should be a part of their interface? This paper presents the results of the focus groups and shows the critical importance of engaging with participants regarding any proposed system changes.

## Methods

Focus group participants were recruited from the EXCEED Public and Participant Engagement Group. The EXCEED cohort, which has consent for re-contact of participants, sent out information on this study to its members; those who were interested contacted JM directly to agree a date on which they could attend. JM had previously presented the concept of dynamic consent and the fact that the research was going to be conducted at an earlier Public and Participant Group meeting. This was an impartial presentation to tell members that EXCEED was investigating this idea and wanted the input of those interested.

Both researchers have had prior experience in conducting qualitative interviews and focus groups. SEW is a consultant academic researcher, who was previously Lecturer at the University of Leicester; She led the focus groups discussions. JM was, at the time of research, Professor of Law at the University of Leicester and is now Professor of Law at the University of Leeds. He assisted with leading the discussions and took written notes.

All FGs were audiotaped with consent of the participants. Audiotapes were transcribed and transcripts anonymised. The transcripts were not returned to participants for checking. Thematic analysis was chosen to analyse the data. Both authors independently reviewed the written transcripts. SW created the initial codes from the data (no formal coding tree was prepared) and from these, suggested initial overarching themes. JM compared these to the themes he had identified independently. Both authors conferred and then agreed the thematic descriptions. SW drafted the first selections of illustrative quotes. JM revised and added additional quotes. All audio tapes were destroyed after transcription. The signed consent forms and transcriptions will be retained in a secure location for six years and then destroyed, in accordance with University of Leicester’s Information Handling Policy. The results were presented and discussed at an EXCEED Public and Participant Group meeting in November 2019.

## Results

Four focus groups, with 3–6 men and women in each, were held at College Court, University of Leicester, in a private meeting room over two days in June 2019. Each session lasted approximately 1–1.5 h with a break, if desired, offered halfway through. Two participants did not attend on the day with no reason given, so one group only had one participant; this was conducted as an in-person semi-structured interview. Only the participants and the two researchers were present at each focus group. At the beginning of each session, participants were welcomed and told how the session would proceed. They were told that they could leave at any time if they wished without explanation. They were asked for their consent to have the session audiotaped. Consent forms were passed out and participants asked to complete them. Travel expenses were covered for participants to attend and forms for this were also distributed.

Focus group members were given an overview of the EXCEED Study and then of specific elements of dynamic consent. For each element, we sought to gauge their understanding of the concept, link this to related experiences and explore their reactions and comfort levels (study interview guide available as a supplementary document). The goal was to map out what a dynamic consent interface might look like for these EXCEED participants.

### Overview discussion of the EXCEED Cohort Study and introduction to dynamic consent

Each focus group began with an overview of EXCEED to remind the participants of its aims and the details of the consent under which they had agree to participate. Dynamic consent, as a concept and as an interface, was also next introduced. They were introduced to the fact that a dynamic consent interface could help participants understand better what data EXCEED had about them. They could, for example, check to see if it was up to date and change it if needed. Participants were positive about this:

I never even thought – or probably didn’t even know how to get in touch with to tell you of my change of address, so something like that I think is quite, I would say is useful. [Female 1, Focus Group 2].

It also became clear that many of our participants had difficulty remembering what their participation in EXCEED involved. They thought that having a homepage describing EXCEED, its aims and its progress would be a good thing to remind them to what they had consented. This showed that participation in a study can be put to the back of one’s mind:

[I]f you ask me quite what I agreed to I probably couldn’t tell you at the moment. …[A] brief bit of what EXCEED is and…what the agreed research purposes initially were… a reminder would be quite useful. [Female 1, Focus Group 2].

It was during this initial discussion that the issue of the security of data within EXCEED was raised by the participants. Indeed, in two of the groups it was the very first thing mentioned, showing that it held paramount importance to our participants. As they have provided their personal data to EXCEED, including their genetic profile, keeping those data secure was a key requirement for them. There may have been some confusion as some participants appeared to believe that a dynamic consent interface would link directly to their EXCEED data. Even with the reassurance that this would not be the case, participants reiterated their view that a secure system was vital for retaining their trust in EXCEED and their participation:

And the most important part of it would be the security, the encryption….I go to lots of different things in the NHS, the people I talk to, they’re scared of that that information is going to get out….I’ve said ‘why don’t you do the research’, ‘oh I’m not giving them my details.’ [Male 1, Focus Group 1].

[A]nything I give you that’s going to go on the internet is not 100% safe…so the next stage is what realistically will be in place to protect any that that’s going to be accessible in that way, and do I think what you’re taking from me matters so much that if it did get breached, how upset would I be about it… [Female 1, Focus Group 2].

### Ability to provide additional information about oneself and see how data was being used

Participants next discussed whether they would like to be able state their preferences around adding new data, for example through online questionnaires. This could include saying ‘yes’ or ‘no’ to being asked about or agreeing to some approaches from outside sources. Many said that they completed questionnaires for other studies and would be happy to do this for EXCEED, although they preferred it if the questionnaires were easy and fast to complete:

Yeah, I think it would be quite easy for you to set it up anyway. And then of course collecting the data is going to be a lot easier for you because it’s going to be the computer and not only that, as long as you say it’s yes/no or there’s three choices. If you start getting where people have to type things out – … [Male 1, Focus Group 1].

Very much so, if it’s multi choice answers, not as…over there said, your life story” [Male 4, Focus Group 1].

But as this participant went on to say, they also wanted know results from the research to which they were contributing:

I’m fed up with sending questionnaires and not knowing what’s going on when it’s finished. [Male 1, Focus Group 1].

It was clear that the participants were eager to have whatever results from research that might help improve their or their family’s’ health or well-being, as was shown in this exchange:

[Male 2, Focus Group 3] “I would be open in wanting to know everything –

[Female 2, Focus Group 3] So would I.

[Male 2, Focus Group 3] About myself, because if you can do something that’s going to stop, or make something slightly easier, not quite so harsh, I don’t know…, but if there’s something that could ease or help, then yes, I’d want to know.”

Participants also wanted to be able to ignore an interface if they wished. They welcomed the thought that they could take a vacation from it, notifying EXCEED if they were to be out of touch for a length of time, but would be happy to be contacted again on their return:

[S]ome people wouldn’t want to be contacted at all, or might say that this is getting too much and would say ‘I’d like to opt out for a while’,…and then could change …when they felt they did want to join in again. [Female 2, Focus Group 1].

But they would not welcome a complicated system or one that required significant attention:

…I’m a bit worried…are you getting too complicated. …Have a safe, well put together IT system and keep the questions simple and relevant. And then we’ll carry on being part of the study. [Female 1, Focus Group 2].

How will we know that there’s a question on this site, presumably they’ll email us won’t they, to say you need to go on to your site. We’re not expected to go on it every day to see if anything’s… [Male 1, Focus Group2].

Opinions differed regarding how often would be burdensome, with the general feeling being:

[p]robably no more than two or three times a year, I don’t know. Something like that [Female 2, Focus Group 2].

### Ability to withdraw completely or for a period

This desire for a system that was easy to use also applied to being able to withdraw. They felt it would be helpful to do this through the interface and to be able to choose levels of withdrawal (e.g., complete destruction of all samples and no further use of data, no further contact but use of their samples and data, etc.) But the interface could also give them a chance to reconsider their withdrawal decision:

You could have gone through a really bad period of things, you know,…’there’s no point in this, I’m out.’ And then a few days later you think again. So, I would think definitely a time lapse period. [Male 2, Focus Group 3].

### Ability to be recruited to other studies

The participants considered how they wished to be recruited to other research studies. There were differing views as to who could contact them for what research purpose. Some people did not mind being approached by a broad range of researchers for differing projects. They recognised that this sharing of data for other research purposes was key:

[B]ecause you don’t know what people want to study…there’s all sorts of different studies going on…and you don’t know whether you can help until you’re asked. [Female 2, Focus Group 3].

There was an almost universal desire that requests come through EXCEED rather than from outside researchers. They had confidence that EXCEED would have vetted those making the request. This was made plain by one participant, who made the point in explicit terms:

I don’t really know whether this drugs company is benign, a nice company, and this drugs company’s horrible, I’ve no way of knowing. But what I would hope is that you would make some judgements about who you wanted to be involved with or who – you know. So again, it comes back to this trust business, doesn’t it…? If I’m involved with the EXCEED study, *I’m happy to be involved with it until I lose faith with the EXCEED study or Leicester University or whatever*. [Male 1, Focus Group 2, emphasis added].

### Ability to receive information about yourself

EXCEED participants had already stated their willingness to be notified of incidental findings as part of the original EXCEED consent. They were happy for the interface to give them the choice of whether they wanted results or not. Participants were clear, however, that if someone wanted to contact them with important news, it should be delivered personally and not through the interface or by letter:

[S]urely you wouldn’t then just send me something that says ‘well actually you’ve got this’, ‘you’re showing that you’re going to get dementia in the next five years’, … surely it would be better to say a general ‘do you want to know, yes or no’, on the understanding that if you do find anything you’re not just going to get a bald letter or whatever…. It’s going [to be] ‘someone is going to contact you and talk you through it’. [Female 1, Focus Group 2].

### Dynamic consent and possible marginalisation

Unprompted, the question of whether dynamic consent might be a disadvantage to some was raised. Two of our participants in different focus groups stated that they either did not use the internet regularly or did not own a computer. This prompted one participant to ask:

[H]ow are you going to deal with people who say ‘I don’t use the internet’ and everything else, … not everyone’s computer literate, so if you go down this route how are you still going to make sure that you don’t exclude people… [Female 1, Focus Group 2].

The possibility of being marginalised also was raised by another participant who noted the low representation of those from the British Asian communities in the EXCEED cohort.

### Security, trust and loyalty

The strongest theme found across the focus groups was that participants trust the EXCEED investigators and the affiliated institutions. They are happy that they joined the study and, providing the current level of security for their data is maintained, remain content with things as things are. They did understand why we were exploring adding an interface:

[Y]ou are now faced with this problem of…data protection… And so obviously you have a lot of thinking to do … and one of the solutions … is to ask us lots of question about what we want to happen. But I think in a way we’ve given you our consent, you know? … [I]t up to you to do the hard thinking and to come up with the solution here. … And we’re quite happy … to say we’ve given you our consent, this is the criteria which we’ve given you our consent under, don’t bother us with lots and lots of questions … [Male 1, Focus Group 2].

Two facets to trust were evident: there was a very high level of trust in EXCEED, and a confidence that EXCEED would not provide inappropriate access to their data:

…I would be happy to have something that pinged to me and said ‘go and have a read’…if I have a request from [City] or wherever,…that they are looking to do this, …are you happy for them to see your data, …and see if it’s something that…I would like to take part in. I would have to trust that you had checked that they were ethical and meeting all the things that – before you even requested it…and I have no reason to believe you wouldn’t be doing that. So, then I can look at it and just think…get on with it. Or, I don’t fancy that one. [Female 1, Focus Group 2].

The participants acknowledged that they had to trust EXCEED to judge the scientific quality and ethical robustness of requests for the use of participant data

Meanwhile, there was a *lack* of trust in an interface if it was linked to their personal data and was not perceived as secure:

Giving you the data,, giving you our name and address and all…how do I know how secure it is at your end? How are you going to convince me that it is secure, because we hear of breaches every day. [Male 1, Focus Group 2].

Thus, in relation to security, the trust and loyalty displayed by participants towards EXCEED was evident. They were happy for EXCEED to use their information as agreed (and were thus content with the broad consent that they had provided) *as a direct consequence* of that confidence. But it was also made clear that this confidence could be eroded:

I only have two concerns really, one is that I can trust the people who are doing it, that’s my first concern. But my second concern is that all these things happen at the moment within a sort of fairly benign sort of social and political sort of culture. And it seems to me that in some ways you can feel that is changing, so what’s it going to be like five years’ time or ten years’ time, you know, what – it’s how things might change. At the moment I feel that I can trust people, you know, and I don’t really care, do what you like, at the moment. I just have slight misgivings that in the future things might be different and universities might come under different sorts of political and social pressure, you know, and that’s already I understand, starting to happen a little bit in [country], and I’m just uneasy about it. [Male 1, Focus Group 2].

Furthermore, this trust was linked to the altruism that motivated them to join the project in the first place:

When I had a stroke, I got the thing in my head that I wanted to help as many people as I can, because I would hate anybody to have to go through what I went through. And anything I can do to help anybody in the future will, and – I will give my full consent to. I’ve got the same sort of opinions. [Male 2, Focus Group 3].

Payback time. [Male 1, Focus Group 3].

## Discussion

In general, our participants thought that a dynamic consent interface would be a good addition to EXCEED. They agreed with many points that had been found in the literature. They felt it would help them to interact with the study and its personnel. They could update address information or how often they might be contacted to add more data. They could state their preferences regarding whether to receive individual research results. They would welcome information on how their data was leading to new research advances. On the other side, our participants did raise points that reflect some concerns about dynamic consent. Some of our participants did not use computers or smartphones, although they did suggest other ways that they might be able to access the interface (i.e., through relatives or by using a shared computer at a library.) Any interface needed to be easy to use and something they could ignore if they wanted. Some aspects of dynamic consent did not arise, such as the suggestion that dynamic consent could improve scientific literacy or empower participants [6]. Similarly, there was no comments that dynamic consent might provoke feelings of scientific inadequacy.

One oft-mentioned aspect of dynamic consent is that it can show respect to participants and improve their trust in a research study[12]. Based on our focus groups, our participants have high levels of trust in EXCEED and are content with their current status. They appeared proud of their contribution and feel a part of EXCEED. They were happy if the researchers wanted to pursue an interface, but they did not give any strong indications that it was needed. If anything, the addition might appear to introduce risk, and might jeopardise their relationship with the study. Contrary to the literature, we believe that the introduction of a dynamic consent interface at this stage would not *improve* participants’ relationship with EXCEED but could introduce a risk of causing a deterioration in trust if something went wrong. In fact, it became apparent that this key issue pervaded all the themes that we have identified. The risks associated with an erosion of trust in EXCEED outweighed any anticipated benefits.

Ethically, there is much to be said for the introduction of dynamic consent interfaces. Unless we espouse a form of libertarianism where research participants ‘sell’ their data or consent as a single process, the broad and blanket consent can only be seen to be suboptimal according to the reasoning behind dynamic consent models. This becomes even more the case the greater distance between the consent given for the original project and any data access requests, given that participants often do not remember to what they originally consented[29]. Indeed if, as the Supreme Court noted, “the courts have become increasingly conscious of the extent to which the common law reflects fundamental values”[4], then the development of dynamic consent can only be considered to be a positive development. A future court could, although we think it unlikely in the near future, ask why one was not incorporated into a research study if a participant were to complain after the fact that their information was used in a way not envisaged when they provided broad consent. After all, the Supreme Court in *Montgomery* also held that the uncertainty for medical professionals caused by a patient-centred legal framework (albeit in relation to physical harm) could be justified if the aim were to protect the interests of patients: “a degree of unpredictability can be tolerated as the consequence of protecting patients from exposure to risks of injury which they would otherwise have chosen to avoid. The more fundamental response to such points, however, is that respect for the dignity of patients requires no less”[4].

Our findings suggest that there could be potential advantages to using a dynamic consent interface for *certain* groups, but not for others. For instance, patient and rare disease communities appeared especially enthusiastic. Therefore, one potential avenue for future research would be to investigate whether there are contextual differences that might make some studies appropriate for an interface but not others. For example, could the makeup of the cohort and its stage of development be key factors in deciding whether to have a dynamic consent interface? It may be found that using the interface with single-site studies from the beginning might increase success. Attempting to retrofit a healthy volunteer study that has been progressing safely and with positive results, such as the EXCEED study, may not be a useful exercise. But more empirical evidence in all these settings is needed.

We also feel that the study highlights the need for some more legal research in this area. Within the focus groups, as noted above, we found a range of views about a dynamic consent interface, but almost universal satisfaction with the broad consent that had been provided, a function of the trust in EXCEED. Of course, cases only come to court when that trust has been eroded or broken, and the question becomes how the law might respond where broad consent was provided but the relationship changed. This includes the question of how it *ought to* respond, particularly when some participants will have been happy with whatever happened to break the bond between researchers and some participants. These are complex issues of policy as well as detail and, as we suggested earlier, they are exacerbated by the courts’ current view that some uncertainty on the part of medical professionals may be warranted in return for greater protection of the dignity of patients. One solution might be to insist on a dynamic consent framework so that researchers could protect themselves legally. Given what we have found above this might seem almost like imposing the model against the wishes of participants. A more detailed legal analysis than there is room for in this paper needs to be conducted.

## Limitations

This study has several limitations. It was quite small, and participants were self-selected. Additional focus groups would be needed to ensure representation of views across the cohort. As members of the EXCEED Public and Participant Engagement Group, they are committed to the study. However, because they are interested in outreach processes, it was felt they were an appropriate group to recruit. While some participants were introduced to the concept of dynamic consent and the fact that EXCEED were exploring adding an interface, we do not feel that this biased their views ahead of the focus groups. There are a small but significant number of EXCEED members from the minority South Asian community. One member did participate but a greater number would have been needed to draw any conclusions as to whether dynamic consent might be more or less welcomed by this community. An additional independent researcher would have been useful when analysing the data to confirm the codes chosen, suggest themes that might have been missed and provide added impartiality to the analysis process.

## Conclusions

Dynamic consent has been mooted as having, “…the potential to radically change the nature of participation in both clinical care and research”[6]. Success has been shown when used in the rare disease and other patient communities. There is more limited evidence for its effectiveness in healthy volunteer biobanks. While it is argued that dynamic consent interfaces can be beneficial for improving engagement with minority populations[15], more evidence is needed.

We found our hypothesis disproven. While in general positive about the concepts around dynamic consent, our participants did not appear eager to have a dynamic consent interface as part of the existing EXCEED study. While they were ready to give suggestions as to the characteristics they felt should be a part of the interface, they did not strongly advocate for one. Our findings also raise the question of whether the addition of an interface might introduce more risk to the trust held by the participants than provide benefits. Our participants were content with the broad consent that they had given, in large part due to the trust that they had in EXCEED. Additional research is needed to further explore whether dynamic consent is needed by all, or whether there are specific contexts where it would be most welcomed.

## Data Availability

The data used and/or analysed during the current study are available from the corresponding author on reasonable request.

## Abbreviations

CHRIS: Cooperative Health Research in South Tyrol
GDPR: European General Data Protection Regulation
EXCEED: Extended Cohort for E-health, Environment and DNA
RUDY: Rare UK Diseases of Bone, Joints and Blood Vessels Study
